# Exponential Fusion of Interpolated Frames Network (EFIF-Net): Advancing Multi-Frame Image Super-Resolution with Convolutional Neural Networks

**DOI:** 10.3390/s24010296

**Published:** 2024-01-04

**Authors:** Hamed Elwarfalli, Dylan Flaute, Russell C. Hardie

**Affiliations:** 1 Department of Electrical and Computer Engineering, University of Dayton, 300 College Park, Dayton, OH 45469, USA; elwarfallih1@udayton.edu (H.E.); flauted1@udayton.edu (D.F.); 2 Applied Sensing Division, University of Dayton Research Institute, 300 College Park, Dayton, OH 45469, USA

**Keywords:** multiframe super-resolution, convolutional neural network, fusion of interpolated frames, image restoration, subpixel registration

## Abstract

Convolutional neural networks (CNNs) have become instrumental in advancing multi-frame image super-resolution (SR), a technique that merges multiple low-resolution images of the same scene into a high-resolution image. In this paper, a novel deep learning multi-frame SR algorithm is introduced. The proposed CNN model, named Exponential Fusion of Interpolated Frames Network (EFIF-Net), seamlessly integrates fusion and restoration within an end-to-end network. Key features of the new EFIF-Net include a custom exponentially weighted fusion (EWF) layer for image fusion and a modification of the Residual Channel Attention Network for restoration to deblur the fused image. Input frames are registered with subpixel accuracy using an affine motion model to capture the camera platform motion. The frames are externally upsampled using single-image interpolation. The interpolated frames are then fused with the custom EWF layer, employing subpixel registration information to give more weight to pixels with less interpolation error. Realistic image acquisition conditions are simulated to generate training and testing datasets with corresponding ground truths. The observation model captures optical degradation from diffraction and detector integration from the sensor. The experimental results demonstrate the efficacy of EFIF-Net using both simulated and real camera data. The real camera results use authentic, unaltered camera data without artificial downsampling or degradation.

## 1. Introduction

In most image acquisition scenarios, there is a desire for the highest spatial resolution possible. However, ultimately, image resolution is limited by the sensor technology and characteristics. Using image post-processing techniques, it is possible to mitigate some of the limitations of a given sensor that may include noise, blur, and aliasing from undersampling. Processing that seeks to improve the resolution of an image beyond that of the native sensor is referred to as super-resolution (SR) [1]. Methods that use one input low-resolution (LR) image and produce one output high-resolution (HR) image are referred to as single-image super-resolution (SISR), while methods where multiple frames are fused to produce one HR image are referred to as multi-frame SR (MFSR). Several significant works documented in the literature [2,3,4,5,6,7,8] address SR for high-dimensional inputs, such as videos and 3D scans. The generation of HR images offers enriched details of locations and inherent objects, proving crucial in various applications, including high-definition TV sets, larger computer screens, and portable devices, like cameras, laptops, and mobile phones.

Convolutional neural networks (CNNs) have successfully been applied to many image processing and analysis tasks in security, surveillance, satellite, and medical imaging [9,10,11,12,13,14,15,16,17]. Early CNN-based SISR methods were introduced by Dong et al. [18,19]. Kim et al. presented the VDSR and DRCN networks [20,21]. Tai et al. pioneered DRRN and introduced memory blocks in MemNet [22,23]. Furthermore, SRGAN, a GAN-based approach for photo-realistic SR, was proposed by Ledig et al. [11]. ResNet was introduced by K. He et al., which was later extended to SRResNet [24]. EnhanceNet utilized a GAN-based model to merge perceptual loss with automated texture synthesis [14]. Recent years have seen significant improvements in deep SISR algorithms, such as EDSR [13], wide feature models by Yu et al. [25], and the residual channel attention network (RCAN) by Zhang et al. [26].

In contrast to SISR, MFSR focuses on extracting information from multiple LR images of the same scene, proving effective in overcoming undersampling issues in imaging systems [1]. In cases with relative motion between the scene and camera during video acquisition, subpixel displacements are common between frames. The diversity in sampling due to this motion can be leveraged to increase the sampling rate, reducing aliasing and improving resolution [27]. With MFSR, there is a trade-off between the temporal and spatial resolution [28]. Deep learning-based approaches have recently been applied to MFSR problems [29,30,31,32,33,34]. Bhat et al. proposed a multi-frame burst SR method that uses PWCNet for feature alignment and an attention-based fusion mechanism [35]. Notably, deformable convolution has proven effective in addressing interframe alignment issues [36,37,38,39]. Several recent architectural advancements have been proposed for specific purposes. For example, the work by Cao et al. [40] focuses on enhancing the resolution of human facial images. This approach involves extracting distinctive features from each LR image and utilizing these features, along with the relative shifts among LR images, to reconstruct the final SR image. In another study by An et al. [41], deep learning methodologies are applied within satellite and remote sensing domains. Additionally, there have been endeavors to integrate SISR and MFSR approaches. For instance, Gonbadani et al. [42] proposed an optimization-based method leveraging LR images and their associated semi-HR images generated via SISR. This optimization yields a closed-form solution, constituting a weighted combination of LR and semi-HR images. A multiframe network has also been introduced by the current authors that uses a single CNN architecture to fuse and restore multiple interpolated input frames [43].

This paper introduces a novel MFSR CNN model, named the Exponential Fusion of Interpolated Frames Network (EFIF-Net). The EFIF-Net extends the authors’ previous algorithm, the Fusion of Interpolated Frames Network (FIFNET) [43]. This new model seamlessly combines multi-frame image fusion and restoration within a single end-to-end network. Key enhancements in EFIF-Net include the introduction of a custom exponentially weighted fusion (EWF) layer for image fusion and a modification of the RCAN for restoration to reduce the blurring in the fused image. We employ subpixel affine motion registration that accounts for camera platform motion. The input frames are externally upsampled and aligned using single-image interpolation. The interpolated frames are then fused using the custom EWF layer, taking into account subpixel registration information to assign more weight to pixels with lower interpolation errors.

For the generation of training and testing datasets, we simulate realistic image acquisition conditions, incorporating ground truth data. Our observation model captures optical degradation from diffraction and detector integration in the sensor. The experimental results highlight the effectiveness of EFIF-Net, using both simulated and real camera data. Notably, the real camera results are based on unaltered, authentic camera data without artificial downsampling or degradation. Owing in large part to the newly introduced EWF layers, the EFIF-Net method surpasses the performance of the previous FIFNET, particularly when dealing with a higher number of input frames.

The remainder of this paper is organized into several sections as follows. In Section 2, we define our observation model and introduce the proposed EFIF-Net MFSR method. We also provide details about network training and the performance analysis conducted. Experimental results are presented in Section 3. Finally, we conclude the paper with a discussion in Section 4.

## 2. Materials and Methods

### 2.1. Observation Model

The observation model is a mathematical model that captures the nature of the degradation processes that occur during image acquisition. The observation model can be used to generate synthetic datasets with degraded images and corresponding ground truth images for deep learning. In order to ensure that a CNN can provide a useful SR solution, the degradation process applied to generate training data must imitate realistic degradation effects. In this work, the observation model relates a static 2D ideal scene image with a group of LR observed frames with affine motion, blur, undersampling, and noise.

**Figure 1:**
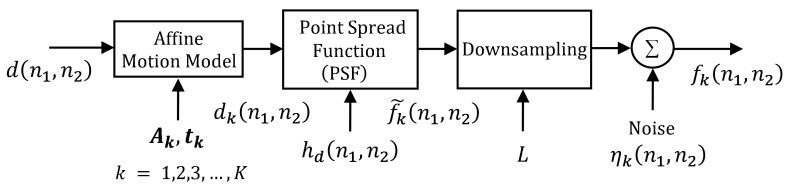
Observation model used to generate training data with known ground truth, as well as testing data for quantitative performance analysis. The input of the observation model is a single HR image $d(n_{1},n_{2})$, and its output is a sequence of *K* LR images $f_{k}\left(n_{1},n_{2}\right)$.

A block diagram of our observation model is shown in Figure 1. This is similar to the one used in the author’s prior work [43], except here we incorporate an affine motion model rather than simple translation as before. The affine model makes the current approach more widely applicable to realistic camera platform motion [44]. The observation model replicates the physical image acquisition process by starting with a Nyquist sampled desired 2D fixed scene $d(n_{1},n_{2})$. Next, the model uses affine warping with bicubic subpixel interpolation to model the relative motion between the camera and scene (usually the result of camera motion and static scene). The affine coordinate warping may be expressed as

(1) $$\tilde{x}\left(k\right)=A_{k}x+t_{k},$$

where $x={\left[x,y\right]}^{T}$ are the reference spatial coordinates and $\tilde{x}\left(k\right)={\left[\tilde{x}\left(k\right),\tilde{y}\left(k\right)\right]}^{T}$ are the warped coordinates for frame *k*. Note that the rotation, zoom and shear for each frame relative to a reference are captured in the matrices

(2) $$A_{k}=\left[\begin{array}{cc} A_{1,1}\left(k\right) & A_{1,2}\left(k\right)\\ A_{2,1}\left(k\right) & A_{2,2}\left(k\right) \end{array}\right],$$

and translation is captured in $t_{k}={\left[t_{x}\left(k\right),t_{y}\left(k\right)\right]}^{T}$, for k=1,2,...,K. The output of the warping process is represented as $d_{k}\left(n_{1},n_{2}\right)$ as shown in Figure 1.

The next step involves applying a realistic imaging-system point spread function (PSF), $h_{d}\left(n_{1},n_{2}\right)$, using 2D convolution to create the blurred frames $\tilde{f_{k}}\left(n_{1},n_{2}\right)$. The PSF modeling follows that reported previously by the authors [43] using the optical parameters in Table 1. The blurred image is then downsampled keeping every *L*’th pixel in the horizontal and vertical dimensions to produce a below Nyquist rate sampled image. This downsampled image is then corrupted with additive Gaussian noise, resulting in a set of *K* LR frames denoted by $f_{k}\left(n_{1},n_{2}\right)$, where k=1,2,…,K. While this one set of camera parameters is employed here, our observation model can be readily adapted to any camera, given the basic optical parameters of that system.

### 2.2. EFIF-Net Multiframe Super-Resolution

In this section, we present the EFIF-Net MFSR architecture and describe the preprocessing steps. Furthermore, we introduce and explore the significance of our unique innovation, the custom EWF fusion layer. This layer plays a pivotal role in seamlessly fusing interpolated frames, significantly enhancing the overall efficiency and performance of our model. Finally, we detail the network training process.

#### 2.2.1. EFIF-Net Architecture

**Figure 2:**
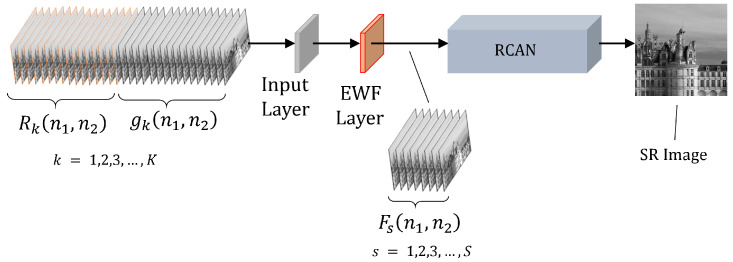
EFIF-Net architecture. Interpolated and aligned observed frames are combined with the subpixel registration information to form the input channels. The red layer represents our custom fusion layer. The fused feature images are then processed with a non-upsampling RCAN network to perform restoration. The output of the EFIF-Net is a single SR image.

The EFIF-Net model is an end-to-end MFSR method as shown in Figure 2. The input is composed of the upsampled and aligned interpolated frames $g_{k}\left(n_{1},n_{2}\right)$ and the corresponding subpixel registration arrays $R_{k}\left(n_{1},n_{2}\right)$ for k=1,2,…K. More will be said about the preprocessing required to produce this input in Section 2.2.2. The EFIF-Net output is one SR image estimate. We design the EFIF-Net model to accomplish two main aims: fusion and deconvolution. The fusion stage is completed by our custom EWF layer that is described in detail in Section 2.2.3. For deconvolution, we employ a modified version of the RCAN model. The RCAN model is a state-of-the-art CNN architecture originally designed for SISR [26]. In particular, we use only the residual in residual (RIR) structure of RCAN to form a very deep network to deconvolute/restore the imagery to produce the final SR image estimate. We remove the upscaling module from the RCAN network because the input size of the network matches the size of its output.

#### 2.2.2. Preprocessing

We preprocess the frames generated by the observation model depicted in Figure 1 using MATLAB Version R2022b to prepare it as the input for EFIF-Net. The LR frames, denoted as $f_{k}\left(n_{1},n_{2}\right)$ and produced by the observation model, are individually subjected to upsampling and alignment using MATLAB’s “interp2” function with bicubic interpolation. This step brings them onto a common L× upsampled HR grid. To automatically determine the necessary affine warping for alignment, we employ subpixel affine image registration using the method in [45], with the initial frame serving as the reference. These individually interpolated frames are represented as $g_{k}\left(n_{1},n_{2}\right)$, where *k* ranges from 1 to *K*. Bicubic interpolation is chosen due to its ability to strike a balance between computational efficiency and performance as outlined in previous research by Hardie et al. [46].

**Figure 3:**
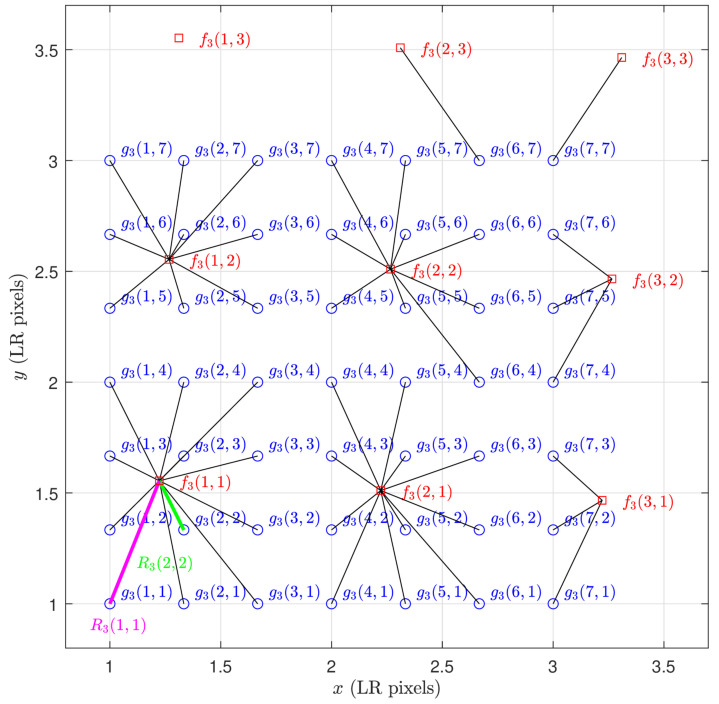
Spatial sampling grid shown in LR pixel spacings. The pixel positions of interpolated frame $g_{3}\left(n_{1},n_{2}\right)$ are shown as blue circles for L=3. The corresponding LR frame samples $f_{3}\left(n_{1},n_{2}\right)$ are shown as red squares for a shift of $s_{3}={\left[0.18,0.6\right]}^{T}$ LR pixels and rotation of θ=−2.527 degrees. The subpixel distances $R_{3}\left(n_{1},n_{2}\right)$ are shown as black lines. An example of a large distance value is shown in magenta, and a small one is shown in green.

Furthermore, we compute subpixel interframe registration information denoted as $R_{k}\left(n_{1},n_{2}\right)$ for each interpolated pixel and introduce these data as additional input channels to the neural network. The distance computation is given by

(3) $$R_{k}\left(n_{1},n_{2}\right)=\sqrt{d_{x}^{2}\left(k,n_{1},n_{2}\right)+d_{y}^{2}\left(k,n_{1},n_{2}\right)},$$

where $d_{x}\left(k,n_{1},n_{2}\right)$, and $d_{y}\left(k,n_{1},n_{2}\right)$ are the horizontal and vertical distances, respectively, of interpolated pixel $g_{k}\left(n_{1},n_{2}\right)$ to the nearest LR pixel in the *k*’th input frame. A visual representation of Equation (3) is provided in Figure 3.

**Figure 4:**
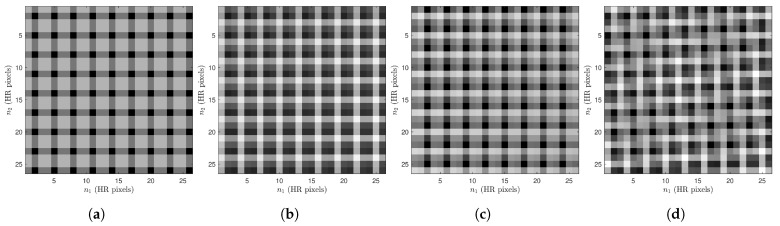
Visualization of $R_{k}\left(n_{1},n_{2}\right)$ for a 25×25 patch size with different shifts and rotations for four frames and L=3. The pixel brightness is proportional to the interpolation distance and presumed interpolation error. Pixel shifts and rotation are (**a**) $s_{1}={\left[0.00,0.00\right]}^{T}$, $\theta_{1}=0$, (**b**) $s_{2}={\left[0.52,-0.40\right]}^{T}$, $\theta_{2}=-0.37$, (**c**) $s_{3}={\left[0.93,-1.08\right]}^{T}$, $\theta_{3}=-0.48$, and (**d**) $s_{4}={\left[0.83,1.28\right]}^{T}$, $\theta_{4}=-4.46$.

Note that the interframe registration information in Equation (3) has the same dimensions as the interpolated frames and may be viewed as images. This is illustrated in Figure 4 for K=3 frames with shift and rotation frame motion and L=3. In this figure, the darker pixels correspond to pixels with smaller interpolation distances, and therefore (presumably) lower interpolation error [44]. We designate the x- and y-shifts in LR pixels for frame *k* as $s_{k}$ and the rotation in degrees as $\theta_{k}$. Figure 4a corresponds to the interpolated reference frame with no motion (i.e., $s_{1}={\left[0.00,0.00\right]}^{T}$ and $\theta_{0}=0$). Here, every *L*’th pixel starting from pixel $n_{1}=n_{2}=1$ lines up exactly with an input pixel and has an interpolation distance of 0. We present three example patches of subpixel registration arrays with motion in Figure 4b–d. They correspond to frame motions of $s_{2}={\left[0.52,-0.40\right]}^{T}$, $\theta_{2}=-0.37$, $s_{3}={\left[0.93,-1.08\right]}^{T}$, $\theta_{3}=-0.48$, and $s_{4}={\left[0.83,1.28\right]}^{T}$, $\theta_{4}=-4.46$, respectively.

#### 2.2.3. Exponential Weighted Fusion Layer

**Figure 5:**
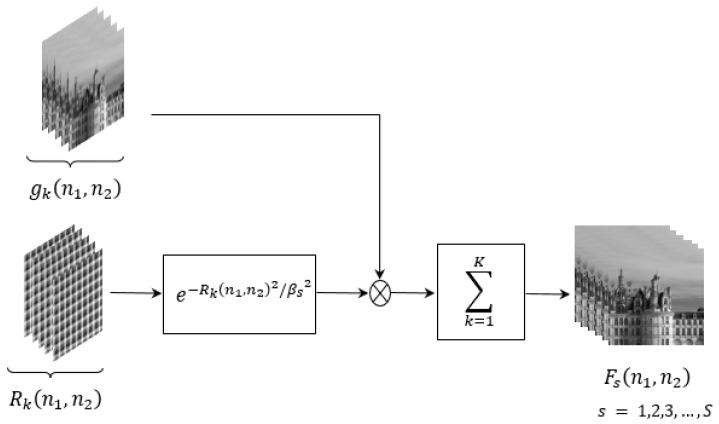
The EWF layer combines interpolated frames $g_{k}\left(n_{1},n_{2}\right)$ with subpixel registration information $R_{k}\left(n_{1},n_{2}\right)$ across various values of the parameter vector β to yield fused frames denoted as $F_{S}\left(n_{1},n_{2}\right)$ for s=1,2,…,S as given by Equations (4) and (5).

In our proposed EFIF-Net architecture, the custom EWF layer is located immediately after the input layer as shown in Figure 2. The EWF layer produces linear combinations of the input frames (fusion), pixel-by-pixel, with weights determined by a decreasing (negative-exponential) function of an interpolated frame’s distance to the nearest measured sample point $R_{k}\left(n_{1},n_{2}\right)$. That is, the EWF layer takes in the *K* interpolated and aligned input frames $g_{k}\left(n_{1},n_{2}\right)$ along with the per-frame subpixel distance arrays $R_{k}\left(n_{1},n_{2}\right)$ and outputs *S* different fused frames $F_{s}\left(n_{1},n_{2}\right)$ as shown in Figure 5. Specifically, the fused images are given by

(4) $$F_{s}\left(n_{1},n_{2}\right)=\frac{\sum_{k=1}^{K}w_{k,s}\left(n_{1},n_{2}\right)g_{k}\left(n_{1},n_{2}\right)}{\sum_{k=1}^{k}w_{k,s}\left(n_{1},n_{2}\right)},$$

for s=1,2,…,S, where the fusion weights are defined as

(5) $$w_{k,s}\left(n_{1},n_{2}\right)=e^{-R_{k}{\left(n_{1},n_{2}\right)}^{2}/\beta_{s}^{2}}.$$

The basic idea behind the EWF layer is that an interpolated pixel that is near an original LR pixel will have less interpolation error and should be given a higher weight in estimating the true intensity of the pixel based on the noisy interpolated intensities. This takes advantage of the multiframe setting since, if diverse camera motion is present, each HR interpolated pixel is likely to have an LR pixel nearby in at least one of the input frames. The rate of decay of the exponential with respect to distance $R_{k}\left(n_{1},n_{2}\right)$ is a hyperparameter $\beta_{s}$ that we control independently for each output fusion frame $F_{s}\left(n_{1},n_{2}\right)$. Note that the fusion process produces a stack of fused frames, each with a different $\beta_{s}$, and these are used as input channels for RCAN restoration. Let us define the vector of all of these EWF hyperparameters as $\beta ={\left[\beta_{1},\beta_{2},\ldots ,\beta_{S}\right]}^{T}$. The optimal choice of β is unknown. But since the EWF layer is differentiable with respect to β, we can optimize β via gradient descent as a learnable parameter of the EFIF-Net model during training. We randomly initialize these parameters and update them during the training process.

#### 2.2.4. Network Training

For training the EFIF-Net model, we use Python Version 3.8 with the PyTorch machine learning framework. The RCAN network model is modified from the publicly available version found in [47]. All pre- and post-processing is performed using MATLAB.

We chose images in the publicly available DIV2K Training dataset, which consists of 800 RGB images of 2K resolution [48]. These images are used as ground truth SR imagery. We converted all the images to grayscale with a dynamic range of 0–1. To simulate degradation during training and testing, we used the observation model explained in Section 2.1 and used SR with L=3. Additionally, we added Gaussian noise with $\sigma_{\eta }=0.001$ to prepare the network for simulated testing data. We used affine motion in the observation model to simulate the effects of camera motion in real-world image acquisition systems. In this study, we applied random affine motion to each image in the training and testing datasets using a transformation model that captures translation and rotation. Transformation parameters were randomly generated and applied to each image to simulate camera motion [45]. We then introduced PSF blur and additive Gaussian noise to the transformed image. By adding camera rotation to the observation model, we are able to address more realistic scenarios than translation alone as was done previously [43].

As the training patches are usually small compared to the original image size, we cropped the 2K resolution images to 480×480 sub-images. The size of the sub-images is different from the training patch size, which is 48×48, but this allows us to avoid reading the entire image when only a small part of it is needed. Each of the 800 images in the DIV2K dataset is cropped to create around 8 non-intersecting sub-images, resulting in a total of 6920 training sub-images of size 480×480. To determine the best length of the β vector *S* in the custom EWF layer, we tested various lengths and found that S=7 is the most effective. The β vector is initialized randomly with values between 0 and 1.

We trained the EFIF-Net model using the default settings of the original RCAN network with 10 residual groups and 20 residual blocks as described in Section 4.1 from the original RCAN paper [26]. The batch size was set to 16 patches, and we used the Adam optimizer [49] with a learning rate of $10^{-4}$. The model was trained for 104k updates using the L1 loss metric. To train the network, we used a Windows workstation with an AMD Ryzen 3970X 32-core processor running at 3.7 GHz and equipped with two NVIDIA GeForce RTX 3090 Graphics Processing Units. The workstation was sourced from Exxact Corp., Fremont, CA, USA. The training time varied between 8 h and 10 days, depending on the number of frames (*K*).

### 2.3. Performance Analysis

We evaluate the performance of the proposed EFIF-Net in comparison to benchmark methods. A quantitative performance analysis is conducted using images subjected to simulated degradation. Subsequently, a subjective assessment using authentic, unaltered camera data is conducted to evaluate the model’s real-world performance.

#### 2.3.1. Simulated Data

The imagery used for quantitative testing comes from three publicly available databases. These are the DIV2K Validation dataset [48] (100 images), the Set14 dataset [48] (14 images), and the BSDS100 dataset [50] (100 images). None of the images contained in these databases were used in training. The testing data underwent the same observation motion as the training data described in Section 2.2.4 so that we have objective ground truth for quantitative performance assessment. As benchmarks for the proposed EFIF-Net SR method, we also used single-frame bicubic interpolation, SISR with RCAN [26], and MFSR using FIFNET [43]. We employed two performance metrics, Peak Signal-to-Noise Ratio (PSNR) and the Structural Similarity Index (SSIM) [51]. We also present a number of processed images for subjective evaluation.

#### 2.3.2. Real Camera Data

In addition to the quantitative performance analysis with simulated image degradation, we also applied the EFIF-Net and benchmark methods to real camera data acquired by the authors. The camera is an Imaging Source DMK21BU04 visible USB camera with a Sony ICX098BL CCD sensor with the optical parameters listed in Table 1. The camera is equipped with a 5 mm focal length lens set to an f-number of F=5.6. The interframe motion was generated by manually panning and tilting the camera on a tripod during image acquisition, which was carried out at a rate of 30 frames per second. Using real-world images for testing SR models provides a more realistic evaluation of their performance.

Because the data were not artificially degraded, there are no corresponding ground truth images available for comparison. Thus, the results with real camera data are presented solely for subjective evaluation purpose. To facilitate this process, we selected familiar scene content and a well-defined test pattern. Since the observation model presented in Section 2.1 is based on a realistic camera PSF, the trained network can be applied directly to the data from that camera. However, to better match the observed signal-to-noise ratio for these real camera data, the networks were trained with appropriate values of $\sigma_{\eta }$ defined in Section 3.2.

## 3. Results

The experimental results are presented in this section for the simulated data in Section 3.1 and real camera data in Section 3.2. The details of the experimental procedure regarding pre-processing are given in Section 2.2.2. Network training details are given in Section 2.2.4, and the performance analysis details are provided in Section 2.3.

### 3.1. Quantitative Results with Simulated Data

**Figure 6:**
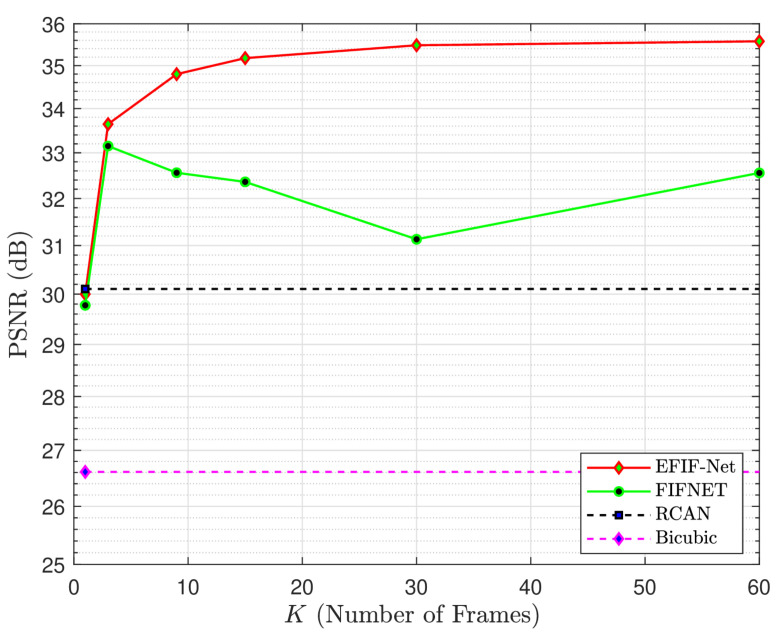
Quantitative performance comparison using simulated data from the DIV2K Validation dataset for L=3 and $\sigma_{\eta }=0.001$. The average PSNR is shown as a function of the number of input frames for the methods shown in the legend.

The graph in Figure 6 shows the average PSNR results for the DIV2K Validation dataset, based on the number of input frames used. The upsampling factor *L* is set to three, and the noise standard deviation is $\sigma_{\eta }=0.001$. The graph shows results for EFIF-Net and benchmark methods. The results in this experiment show that EFIF-Net outperforms all of the benchmark methods for K>1, with similar performance to the single-frame RCAN method for K=1. Furthermore, increasing the number of input frames improves the performance of EFIF-Net. This is demonstrated by the smooth increase in the PSNR performance curve of the EFIF-Net model in Figure 6 as the number of input frames *K* increases. Table 2 presents additional quantitative results, including the PSNR and SSIM values, for all three test databases. The results demonstrate that the EFIF-Net model with K=60 yields the best quantitative performance across all three databases.

**Figure 7:**
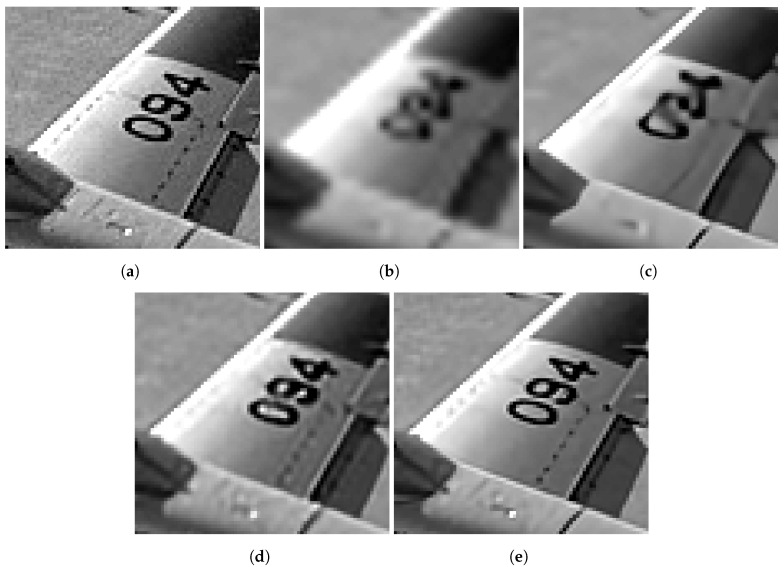
Results for image “071” in the BSDS100 dataset. The PSNR(dB)/SSIM values are (**b**) 26.755/0.726, (**c**) 30.895/0.839, (**d**) 33.183/0.886, and (**e**) 36.558/0.925. The noise has a standard deviation of $\;\sigma_{\eta }=0.001$, and K=60 frames are used in (**d**,**e**). (**a**) Truth, (**b**) Bicubic, (**c**) RCAN, (**d**) FIFNET, (**e**) EFIF-Net.

**Figure 8:**
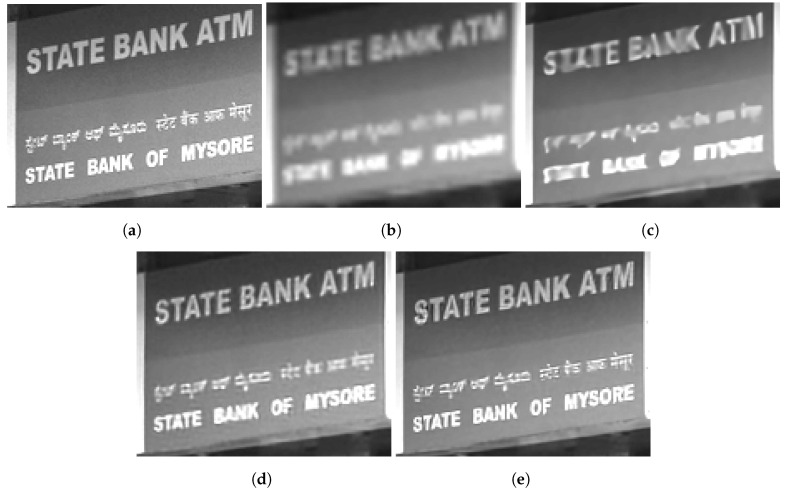
Results for image “91” in the DIV2K dataset. The PSNR(dB)/SSIM values are (**b**) 24.445/0.753, (**c**) 28.279/0.878, (**d**) 30.776/0.928, and (**e**) 34.440/0.961. The noise has a standard deviation of $\;\sigma_{\eta }=0.001$, and K=60 frames are used in (**d**,**e**). (**a**) Truth, (**b**) Bicubic, (**c**) RCAN, (**d**) FIFNET, (**e**) EFIF-Net.

**Figure 9:**
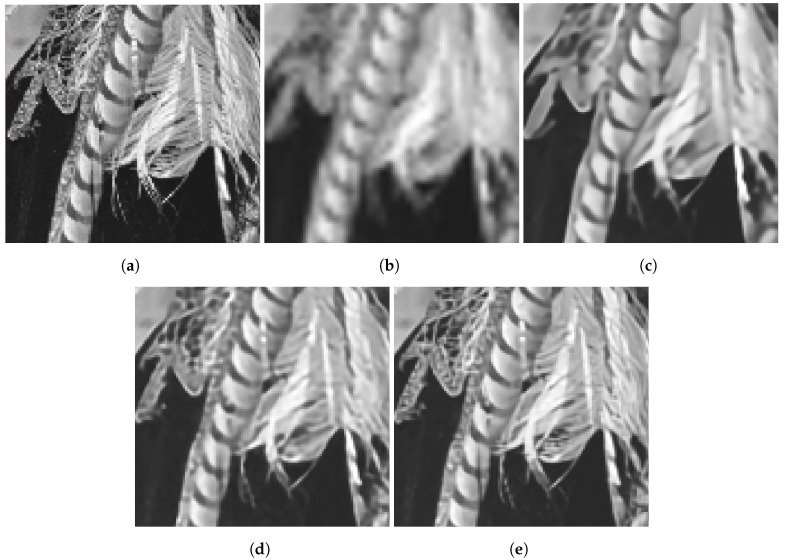
Results for image “10” in the Set14 dataset. The PSNR(dB)/SSIM values are (**b**) 25.025/0.688, (**c**) 27.532/0.796, (**d**) 30.255/0.886, and (**e**) 33.168/0.934. The noise has a standard deviation of $\;\sigma_{\eta }=0.001$, and K=60 frames are used in (**d**,**e**). (**a**) Truth, (**b**) Bicubic, (**c**) RCAN, (**d**) FIFNET, (**e**) EFIF-Net.

**Figure 10:**
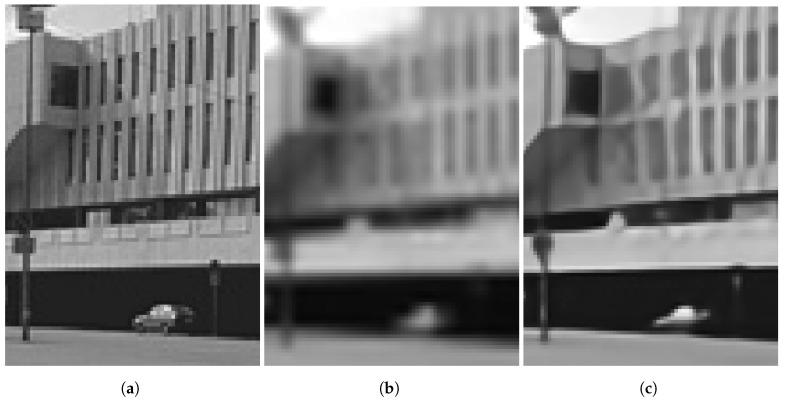

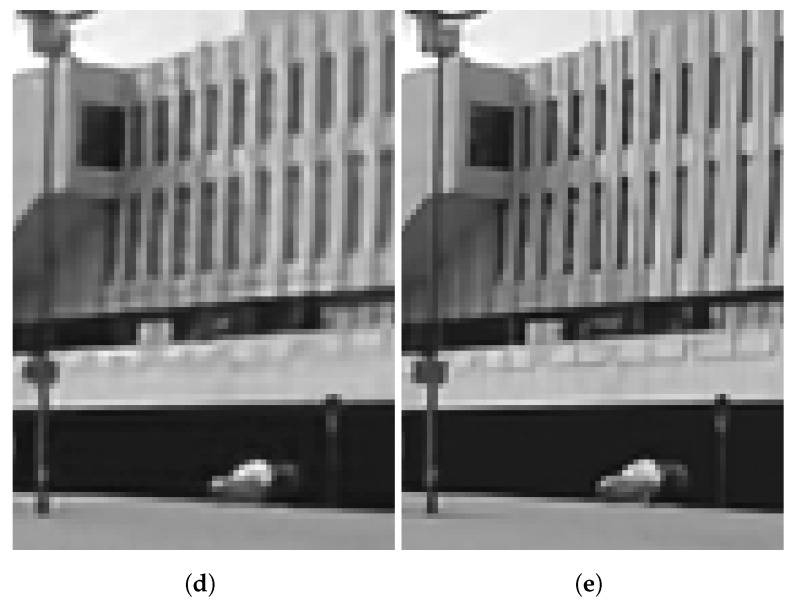
Results for image “92” in the DSBS100 dataset. The PSNR(dB)/SSIM values are (**b**) 25.025/0.688, (**c**) 27.532/0.796, (**d**) 29.318.255/0.889, and (**e**) 34.753/0.960. The noise has a standard deviation of $\sigma_{\eta }=0.001$, and K=60 frames are used in (**d**,**e**). (**a**) Truth, (**b**) Bicubic, (**c**) RCAN, (**d**) FIFNET, (**e**) EFIF-Net.

Figure 7, Figure 8, Figure 9 and Figure 10 provide several processed images for subjective evaluation. Each figure includes the truth image in (a), and the processed ROIs in (b)–(e). It is important to note that the images in (b), and (c) in these figures are for single-frame methods, while the (d), and (e) are for multiframe methods with K=60 frames. The captions of the figures provide the error metric values associated with the images. The image of an airplane in Figure 7 serves as a good example of the typical results. The bicubic interpolation method applied in Figure 7b results in significant blurring and aliasing artifacts in the form of jagged edges along the plane’s wing. Conversely, the RCAN single-frame method applied in Figure 7c performs well in sharpening and reconstructing the edges of the wing. Nevertheless, the ill-posed nature of the inverse problem is evident in the numbers “0”, “9” and “4” in this image. The multiframe methods are relatively successful in recovering these numbers. However, the EFIF-Net result in Figure 7e appears to offer the sharpest images with minimal aliasing artifacts. In addition, the EFIF-Net result in Figure 7e is 3.375 dB higher than the FIFNET model. Similar relative performance results are observed in Figure 8, Figure 9 and Figure 10.

### 3.2. Subjective Results with Real Camera Data

**Figure 11:**
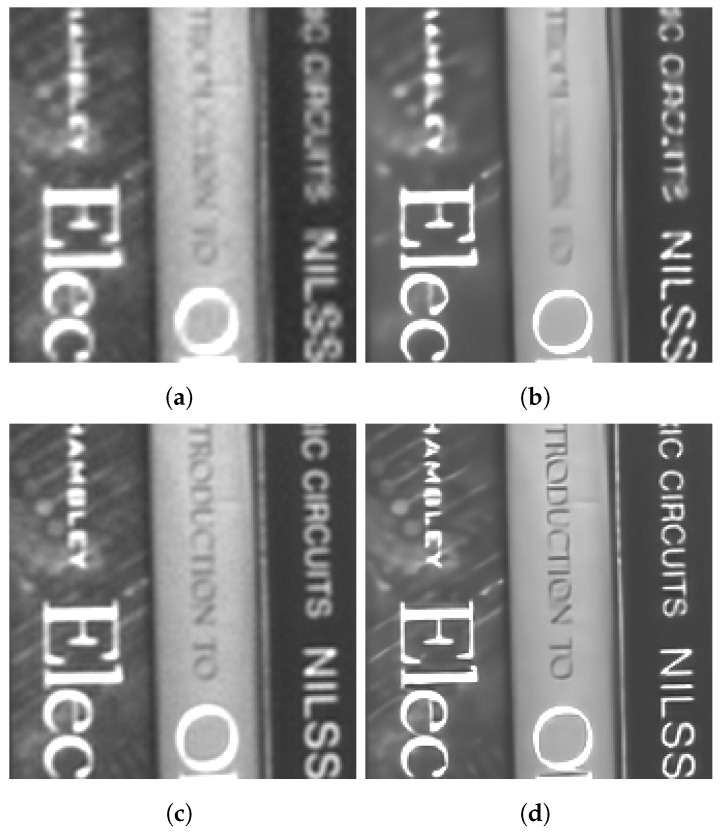
Image results for the real camera data of a bookshelf. The images shown are (**a**) Bicubic, (**b**) RCAN, (**c**) FIFNET and (**d**) EFIF-Net. The noise has a standard deviation of $\;\sigma_{\eta }=0.01$ and K=60 frames for (**c**,**d**).

**Figure 12:**
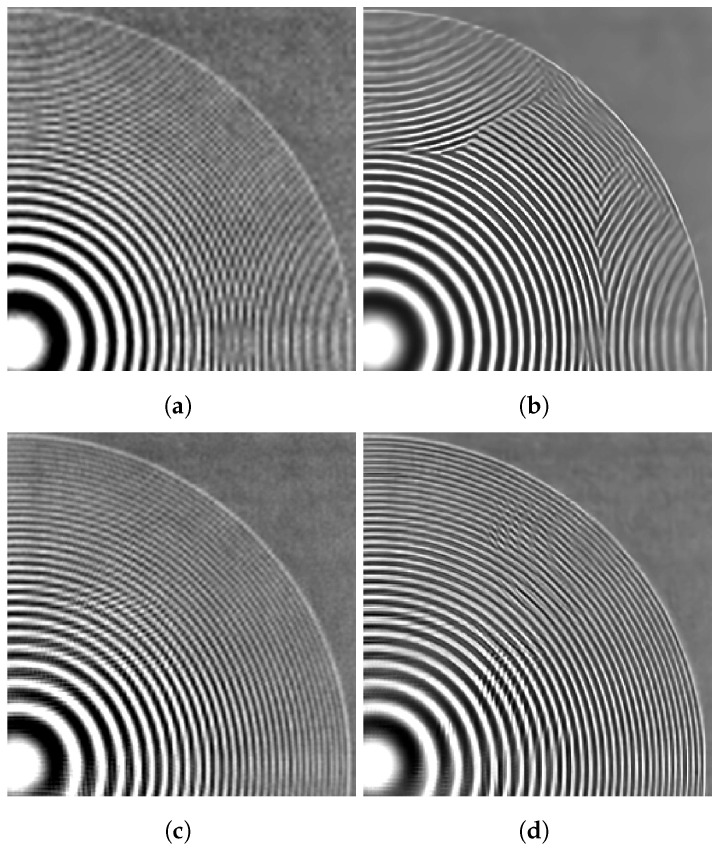
Image results for the real camera data of a chirp. The images shown are (**a**) Bicubic, (**b**) RCAN, (**c**) FIFNET and (**d**) EFIF-Net. The noise has a standard deviation of $\;\sigma_{\eta }=0.025$ and K=60 frames for (**c**,**d**).

We present the results for two distinct real-camera multiframe datasets in Figure 11 and Figure 12. For each dataset, the multiframe methods use K=60 frames with L=3. As mentioned above, these results showcase the potential of the algorithm in a real-world application (and without artificial degradation). Figure 11 illustrates the application of SR models on a bookshelf dataset captured indoors. Note that there is no actual ground truth available for such real images, but the scene content of books on bookshelf is familiar. We selected a value of $\;\sigma_{\eta }=0.01$ for the training noise used for the network that processed these data. In Figure 11a, the bicubic interpolation image exhibits aliasing artifacts on the lettering. Although the RCAN method managed to decrease the aliasing artifacts on larger letters (Figure 11b), it performed more poorly on smaller lettering. On the other hand, the EFIF-Net outcome shown in Figure 11d offers better sharpness and superior noise suppression than that of FIFNET in Figure 11c.

The image in Figure 12 depicts a circularly symmetric chirp pattern that is specifically chosen to demonstrate the ability of the tested methods to reduce aliasing. For these data, we selected a value of $\;\sigma_{\eta }=0.025$ for the training noise level. The bicubic interpolation image in Figure 12a shows aliasing in the form of a Moiré pattern on the high-frequency components of the chirp, causing the concentric ring pattern to appear inverted on the top and right of the image. The SISR RCAN method output shown in Figure 12b increases contrast but is unable to accurately reconstruct the true chirp structure from the undersampled imagery. The MFSR FIFNET method output shown in Figure 12c does show reduced aliasing and resolves more of the concentric rings than single-image bicubic or RCAN. However, the EFIF-Net output in Figure 12d provides the best sharpness and greater noise reduction.

## 4. Discussion

In this paper, we introduced the EFIF-Net approach to tackle the challenge of MFSR using CNNs. Diverging from many previous methodologies, our approach incorporates a realistic observation model in the creation of both the training and testing datasets. This model faithfully accounts for the genuine degradation effects introduced by the camera during image capture. Consequently, we developed a more robust CNN model equipped for real-world scenarios. Furthermore, we enhanced the previous FIFNET in several ways. First, we extended the camera motion model from translation to a more practical affine model. This affine model allows for rotation, scaling, and shear in addition to translation. Rotation, in particular, is important, as it is commonly introduced when acquiring an image sequence from a non-fixed platform. Another innovation in EFIF-Net, that is not in FIFNET, is the inclusion of the custom EWF layer for performing image fusion. Specifically, the EWF layer employs exponential weights to interpolated pixels so as to give more weight to pixels with less interpolation error. Finally, the current method provides superior restoration of the fused image by incorporating a modified RCAN network with many layers, whereas FIFNET uses a relative small CNN.

The experimental results presented in Section 3 demonstrate the performance benefits of the innovations described above. The results show that the MFSR methods provide higher PSNR values than the SISR benchmark method. This shows that the spatial sampling diversity provided by multiple frames, when properly exploited, is a significant benefit to the restoration quality given undersampled input frames. Furthermore, the EFIF-Net consistently outperforms the previous FIFNET in all quantitative and subjective performance analyses. The performance improvement of EFIF-Net is most pronounced with higher numbers of input frames. In fact, the performance of FIFNET initially went down in PSNR when more than four input frames were used. We attribute this drop in performance to the inability of the FIFNET architecture to efficiently fuse large numbers of input frames by simply concatenating them. The EWF layer in the EFIF-Net addresses this issue and reduces the number of learnable parameters associated with image fusion to just the S=7 values in β, compared with a much larger number of learnable parameters associated with the full convolution layers in FIFNET. As a result, the EFIF-Net performance consistently improves with an increased number of input frames as one would hope from an MFSR method. We also observed, in the subjective image results with simulated and real data, that the EFIF-Net provided final results that appeared sharper and less noisy than the benchmark methods. We attribute this to the improved restoration provided by the modified RCAN processing layers, compared with the relatively small number of convolution layers used by the earlier FIFNET.

Our study had limitations. One limitation is that we considered only one camera model due to the significant network training time needed for multiple numbers of input frames and multiple noise levels. Additional performance analyses would be helpful in future work to better understand how the EFIF-Net performs with other cameras and optical parameters. Another important limitation to be aware of relates to the fact the MFSR methods presented here assume a static scene and affine camera motion. The presence of in-scene motion, like moving cars and people, is not addressed in the current study. However, it may be possible to incorporate some scene motion detection and alternative processing [52] in future work. Also, if there is no camera motion, there is generally no spatial sampling diversity. Consequently, the benefits of MFSR over SISR are more limited. Interestingly, the work in [46] demonstrated that mild atmospheric turbulence can provide sampling diversity to improve MFSR, even with a fully stationary camera and scene. Finally, we consider only grayscale images here so as to focus on the essential characteristics of MFSR. However, our approach can be readily extended to color in future work by employing some of the concepts described in [53].

Based on a subjective analysis, the results of the proposed EFIF-Net method using real camera data appear very consistent with the results with simulated data. In the real data, EFIF-Net produced clearly visible aliasing reduction, particularly in the chirp image (designed to illustrate aliasing). It also produced images with increased sharpness and increased noise reduction compared with all of the benchmark methods. Good results on real camera data are particularly notable since the training data were generated using our theoretical observation model and images with simulated degradation. The trained model generalized well from the simulated training data to the real camera testing data that had no artificial degradation, demonstrating robust real-world performance.

The main advantage of the proposed method is the ability to efficiently fuse multiple input frames using the EWF layer in a manner that scales to a virtually limitless number of input frames. In particular, after the EWF layer that produces S=7 fusion images, the rest of the network does not grow in complexity with an increased number of input frames. One important area of future work is to investigate how to address scene motion within the proposed framework in such a way as to preserve moving objects and provide SR enhancement where possible. 

## Figures and Tables

**Table 1:** Optical parameters used for training and testing for real and simulated images. These parameters are based on an Imaging Source DMK21BU04 visible USB camera with a Sony ICX098BL CCD sensor. The camera is equipped with a 5 mm focal length lens set to an f-number of F=5.6. The camera/sensor and optics were obtained from The Imaging Source, LLC, Charlotte, NC, USA.

Parameter	Value
Aperture	D=0.893 mm
Focal length	l=5.00 mm
F-number	*F* = 5.60
Wavelength	λ=0.550 μm
Optical cut-off frequency	$\rho_{c}=324.68$ cyc/mm
Detector Pitch	p=5.6μm
Sampling frequency	1/p=178.57 cyc/mm
Undersampling	M=3.636
Upsampling factor	L=3.000
Noise model	Additive Gaussian ($\sigma_{\eta }=0.001$)
Dynamic range of dataset	0–1
Camera motion model	Affine

**Table 2:** Average PSNR(dB)/SSIM for K=1,9,30, and 60 using different methods and three different datasets: BSDS100, Set14, and DIV2K Validation. The numbers in bold font indicate the best performance for the corresponding metric and dataset category.

Dataset	*K*	PSNR(dB)/SSIM			
		Bicubic	RCAN	FIFNET	EFIF-Net
Set14	1	23.55/0.692	27.46/0.814	27.44/0.808	27.51/0.814
	9	-	-	31.45/0.909	32.67/0.925
	30	-	-	31.16/0.909	33.71/0.939
	60	-	-	31.03/0.903	**34.24**/**0.945**
BSDS100	1	24.31/0.720	26.36/0.781	26.29/0.775	26.36/0.781
	9	-	-	30.11/0.898	31.26/0.918
	30	-	-	29.98/0.897	32.56/0.937
	60	-	-	29.89/0.892	**33.18**/**0.945**
DIV2K	1	26.61/0.790	30.00/0.866	29.69/0.859	29.81/0.864
	9	-	-	33.10/0.919	34.48/0.940
	30	-	-	32.67/0.917	34.97/0.946
	60	-	-	32.41/0.9111	**35.16**/**0.948**

## Data Availability

Publicly available datasets were analyzed in this study [48,50].
